# Injuries of the Head

**Published:** 1828-03

**Authors:** 


### HOSPITAL REPORTS,

(PRINCIPALLY CONDENSED FROM THE VARIOUS PUBLICATIONS.)

### INJURIES OF THE HEAD.

CASE I. *Exfoliation of a Portion of the Temporal Bone, from a Blow.*

J. CLEAVER, æt. eighteen, fell from a scaffolding on the 24th of September: the scalp covering the left parietal bone was torn, and the pericranium partially detached. The integuments were replaced, and some degree of adhesion took place. In the course of the following month, he had two attacks of erysipelas of the scalp, during which he took salines with tartarised antimony, and occasional doses of calomel.

On the 25th of October, he noticed that his right hand was unusually weak. On the afternoon of the 28th, he had an epileptic fit, which lasted five minutes. For the three following days, the weakness of the hand increased; he could move his wrist and fingers, but could not support a moderate weight. The portion of bone which had been originally denuded was found to be exfoliating, a groove having been formed round it by absorption. It appeared likely that at one part or other the exfoliation might occupy the whole thickness of the bone, and that matter had formed upon the dura mater. From this period he gradually recovered the use of his hand. The portion of bone was so loose on the 3d of December as to admit of being removed with the dressings: at the edge it was of the thickness of the outer table only; centrally, for the length and breadth of half an inch, it consisted of the entire substance of the bone. The dura mater and the surface of the living bone were covered with healthy granulations.

The boy soon afterwards recovered entirely; he was treated at the MIDDLESEX HOSPITAL.

CASE II. *Hernia Cerebri.*

Samuel Makeller, six years of age, was kicked upon the temple on the 21st of September: he was stunned by the blow, but, by the time he was brought to the Middlesex Hospital, he had perfectly recovered. The integuments were wounded, and there was fracture with depression, extending across the coronal suture from the parietal to the frontal bone on the right side. The fracture was about three inches in length, the depression of the depth of two lines. The edges of the wound were brought towards each other with adhesive plaster: he was placed upon low diet, and the bowels acted on by aperients, containing calomel, every other night.

For several days he remained perfectly well, but on the 4th of October he became indisposed: he had shivering, and vomited several times; the bowels were confined, and acted upon by medicine with difficulty. The pulse, which had hitherto been 120, fell to sixty, and became extremely irregular, intermitting every third, fourth, or fifth beat. Leeches were applied to the head, and mercurial purgatives were prescribed. The pulse rose, and, though the tongue and skin continued dry, his general appearance was improved. Antimonial salines were administered.

On the 13th, his skin was cool, his tongue moist, his appetite had returned, but his nights were disturbed and restless.

On the 15th, about one o'clock, having had a rigor, he became insensible: the breathing was stertorous, the pulse fell to sixty again, but was regular; the pupil of the right eye was dilated; the right side of the face was convulsed, as well as the left arm and hand. The trephine was applied, so as to allow of the elevation of the depressed portion of bone, which, being found to be detached, was removed. About five ounces of pus escaped from the cranial cavity during and after this operation, that had lodged between the cranium and the brain; the dura mater was in a sloughy state. The child immediately showed signs of returning consciousness: the pupils of both eyes acted perfectly on the admission of light; the pulse rose to 100. Towards evening, he recognised his father and mother.

On the following day, the interval between the bone and the surface of the brain was filled up by a greenish black substance, which rose above the level of the integuments: moderate pressure was made upon it by means of a roller, but the hernia cerebri continued to increase.

On the 19th, a ligature was tied round the base of the tumor, which was then removed with the knife.

By the 27th, the hernia cerebri had again become as large as a walnut, and, though it was again removed, it continued to be reproduced each time till November 1st, when he died.

On opening the head, the arachnoid membrane upon the affected side was found to have become thickened and opaque, and about three ounces of pus were contained between it and the dura mater.

### CASE III. *Concussion, followed by Extravasation.*

William Beacon, æt. thirty, was admitted under the care of Mr. EARLE, at ST. BARTHOLOMEW'S HOSPITAL, on the morning of the 23d of January, labouring under the following symptoms:—Drowsiness, from which he was with difficulty roused; answered questions incoherently; pupils dilated, but obedient to the stimulus of light; pulse small and irregular; extremities cold. There was a lacerated wound of the scalp on the upper and lateral part of the left parietal bone, but the cranium did not appear to be denuded of its periosteum. He was unable to give any account of himself, but the persons who brought him to the hospital stated that he had been thrown out of a cart the night before; he was taken up insensible, and conveyed to a surgeon, who bled him. He soon after recovered his sensibility, and had vomited frequently: this amendment, however, was but temporary, for he soon fell into a state of stupor.

**He was ordered Hydr. Submur. gr ij; P. Jalapæ gr. x. quartis horis.**

At four P.M., reaction having taken place, he was bled to sixteen ounces. At seven P.M. his countenance flushed, his skin hot and dry, with general restlessness and anxiety; pulse 100, full and hard. He was bled to twenty ounces, which produced complete syncope. The head, which had been previously shaved, was kept constantly wet with cold applications. Immediately after the abstraction of blood, the pulse became much softer, and was reduced in frequency; the pupils were less dilated, but acted rather sluggishly.

At twelve P.M. his pulse had increased in hardness, but was not more frequent; he was continually muttering to himself, and appeared very restless.

**V.S. ad ℥xvj—Contr Hyd. Subm. cum Jalapa quartis horis.**

On the 24th, he was violently delirious, and was again bled to sixteen ounces.

25th.—Last evening he was so restless that he could with difficulty be kept in bed; pulse frequent and irregular; the muscles of the left side of the face were now first observed to be drawn to one side. He passed his urine and fæces involuntarily.

**C. C. ad ℥xvj. temporib.—Applicr Emp. Lyttæ. mag. nuchæ statim.**

26th.—More decided symptoms of compression have manifested themselves: the right side of the body is partially paralysed, and there is almost a continued convulsive twitching of the muscles of the face and upper extremity of this side, with impaired sensation. His breathing is not affected, but he is more comatose, and quite inattentive to any one around him. Continues to pass his urine and fæces involuntarily. Upon examining the wound of the scalp, it had not united, and was in a sloughy state; the bone beneath was quite bare to the extent of about an inch, and to all appearance dead; it was quite white, and did not bleed on being scraped.

Mr. Earle considered this one of those perplexing cases which are sometimes met with, where concussion and extravasation are coexistent. In the earlier part of the case, the patient had laboured under the symptoms common to concussion, while latterly those of compression had gradually come on. Considering the active means which had been adopted without any amelioration of symptoms, Mr. Earle conceived it a fit case for the application of the trephine to that portion of bone which appeared to be dead. No blood escaped until the instrument had penetrated the diploe, when about two or three ounces of blood oozed out: the portion of bone was elevated and removed, and the dura mater carefully examined. No blood or matter was found upon its surface, but it had a bluish tint as if blood had been deposited beneath; in other respects it appeared perfectly healthy. The wound bled freely, from one of the branches of the meningeal artery having been opened: a pledget of wet lint was put over it, and the patient conveyed to bed. At nine P.M. his pulse being hard and full, he was bled to twelve ounces.

28th.—He appeared rather more sensible during the early part of yesterday, but gradually sunk, and died last night.

*Sectio cadaveris.*—Blood was found effused underneath the fascia of the left temporal muscle; the dura mater was sound, and free from inflammation. Upon removing this membrane, there was found to be effusion of blood over the whole surface of the cerebrum. On the left hemisphere, exactly opposite where the bone had been removed, a considerable quantity of dark clotted blood was deposited; the hemisphere was much flattened from the pressure. The cellular tissue connecting the vessels of the pia mater was also greatly injected. A fracture was discovered, extending from the upper part of the squamous portion of the left temporal bone, through the superior inferior angle of the left parietal; it extended across the groove of the arteria meningea media. A great quantity of blood had been thrown out between the dura mater and bone, amounting to not less than three or four ounces. The posterior surface of the middle lobe of the hemisphere of this side was coated with a layer of dark grumous blood. Upon making a section of this lobe, its anterior and middle part had undergone that change of structure termed "ramollissement:" it was reduced to a soft cream-like consistence to the extent of about two inches, evidently commencing in that part of the lobe corresponding to the fracture.

Case IV.—*Fracture with Depression, successfully treated without Operation.*

Robert Cockman, æt. thirteen, was admitted at five P.M. Dec. 12th, under the care of Mr. Brodie, at St. George's Hospital, having fallen upwards of twenty feet half an hour previously.

The skin was pale, the pulse ninety-six and feeble. He was perfectly sensible, but had total loss of memory, not even remembering his own name; and upon examination there was found, near the temporal ridge of the left parietal bone, a good deal of extravasation of blood beneath the scalp, and what appeared to be a considerable depression of the cranium.

He had a spirit lotion applied to the head, and was purged.

13th.—Vomited two or three times in the course of last evening, and this morning the pulse has got up to 110, with hot skin, dry tongue, and headache. The bowels have been freely opened.

He was bled to eight ounces, with the effect of relieving the pain in the head; and was ordered salines with antimony every six hours.

From this time no bad symptoms whatever remained. As the extravasated blood was absorbed, the depression of the bone became marked, and no longer bore any resemblance to those cases of pitting in the scalp caused by effusion around the stricken part. The edges were bold and distinct, the depression, we should say, nearly a quarter of an inch in depth, and over it there was an evident fluctuation, but no tumor, the fluid just filling up the hollow. On the 26th, he was made an out-patient.

Sir Astley Cooper, it is well known, has drawn a distinction between simple and compound fracture of the skull, and upon this distinction he has hinged a very material difference of treatment. Mr. Brodie is not inclined to agree with Sir Astley upon this point: first, because he thinks the analogy between fractures of the cranium and fractures of the long bones scarcely a fair one; and, secondly, because the results of his experience have not borne out the distinction.

Case V. *Fracture of the Skull; Malformation of the Brain.*

W. Menzie, æt. seventeen, of weak understanding, and unable, from his infancy, to hold up his head without difficulty, received a blow, about two years ago, on the back of the neck, for which he was for some time a patient at Guy's Hospital. He was brought into St. Thomas's on the 30th of November, having fallen from a window three stories high an hour previously. He was then totally insensible, his skin cold, his face pale, his respiration hurried and laborious; the pupils were contracted, the pulse scarcely perceptible. He had vomited once since the accident. The whole occiput was much bruised and swollen, and, the scalp having been shaved, it seemed as if the posterior superior angle of the right parietal bone was slightly depressed, but there was no other appearance of fracture.

Mr. Travers ordered small quantities of brandy to be administered at short intervals; an enema, containing half an ounce of Ol. Terebinth. to be thrown up; and heated cloths to be applied to the surface of the body.

December 1st.—He has been very restless during the night. Reaction took place yesterday evening, when the brandy was discontinued. The face is flushed, and the skin warmer, but respiration is still very laborious; the pupil contracts and dilates slowly and alternately, apparently uninfluenced by light. The pulse in the left arm is sixty, regular; in the right, it is scarcely perceptible. Pressure on the scalp causes considerable pain. The left arm is motionless, but sensible; the right the reverse. He made an attempt to speak this morning on hearing his mother's voice, but seemed unable to articulate.

Twelve leeches were ordered to be applied to the head, and afterwards a poultice.

3d.—Being still comatose, Mr. Travers made an incision through the scalp, parallel to the sagittal suture, half an inch on the right side of it, and passing posteriorly an inch beyond the lambdoidal suture. The cellular substance beneath the scalp was found infiltrated with blood. The pericranium was closely adherent to the bone, in which no fissure was discernible, and the operation was discontinued. The patient seemed to be somewhat roused by the pain of the incision, and attempted to raise his hand to his head, but soon relapsed into his former condition.

He lingered two days longer, and died at nine in the evening of the 6th of December.

On removing the scalp, the pericranium was found to be thickened in its whole extent from blood effused into its texture; and, when this was separated from the skull, two fissures were perceived commencing from the same point near the centre of the left parietal bone, the one running obliquely across the os frontis, and terminating in its right external orbital process; the other following the coronal suture, quitting it only for the space of about three lines, and terminating at the squamous suture. There was no depression of bone except at the angle made by the two fissures, where a small portion of the inner table was slightly depressed. The dura mater was uninjured, and adherent to the bone, except in the direction of the fissures, where its surface was covered by two or three small coagula. In the same situation, beneath the pia mater, was a more considerable effusion of blood; and, at the edge of the left hemisphere, blood was effused into the cortical substance of the brain, to a small extent. On separating the hemispheres, a large semi-transparent cyst was seen occupying the situation of the corpus callosum, which, together with the fornix, seemed to be wanting, the third ventricle being covered by a thin transparent membrane. This cyst, which was two inches in length, and one in breadth, contained about an ounce of limpid serum, and had numerous small vessels ramifying on its internal surface; it was found to be attached to the left hemisphere immediately above the lateral ventricle, the roof of which it partly formed. This ventricle was greatly enlarged and distended, and contained about four ounces of serum. On taking out the brain, a large coagulum was found on the inferior surface of the right middle lobe, which had a considerable quantity of blood effused into its substance, and was much softer than natural.

The right lateral ventricle was found to be equally distended with the left, the corpus callosum entirely wanting; the fornix was entirely deficient on the left side, and all but its anterior pillar on the right. There was a small hydatid upon the corpora quadrigemina.

---

